# Supplementary material for: Sustainable production of camptothecin from an Alternaria sp. isolated from Nothapodytes nimmoniana
Source: Sci Rep. 2021 Jan 14;11:1478. doi: 10.1038/s41598-020-79239-5 (PMC7809410; doi:10.1038/s41598-020-79239-5)
Supplement: Supplementary file 1 — Supplementary Information [file 41598_2020_79239_MOESM1_ESM.pdf]

Supplementary data

Sustainable Production of Camptothecin from an *Alternaria* sp. isolated from *Nothapodytes nimmoniana*

I. A. H. Khwajah Mohinudeen[1], Rahul Kanumuri[1], K. N. Soujanya[2#], R. Uma Shaanker[2], Suresh Kumar Rayala[1] & Smita Srivastava[1]*

[1]Department of Biotechnology, Bhupat & Jyoti Mehta School of Biosciences, Indian Institute of Technology Madras, Chennai-600 036, India.

[2]School of Ecology and Conservation, University of Agricultural Sciences, GKVK, Bengaluru – 560 065, India

Supplementary Table S1. Yield of camptothecin obtained from the isolated endophytic strains.

S. No	Endophyte	Camptothecin yield (µg/g)	S. No	Endophyte	Camptothecin yield (µg/g)
1	P1N-12-PE4	3.69 ± 0.3	36	P2-8-PE2	0.86 ± 0.1
2	P1N-11-PE1	11.23 ± 2	37	P2-5-LE3	0.56 ± 0.1
3	P1-2-LE3	3.69 ± 0.3	38	P2-6-LE4	1.45 ± 0.3
4	P1N-12-LE2	0.48 ± 0.05	39	P1N-11-PE4	0.28 ± 0.05
5	P2-1-LE3	2.21 ± 0.7	40	P2-7-LE1	12.32 ± 2.6
6	P1N-3-LE2	4.30 ±1.1	41	P2-7-LE3	2.71 ± 0.7
7	P1N-12-PE3	3.33 ± 0.8	42	P2-7-LE2	7.71 ± 1.2
8	P1N-11-PE2	0.21 ± 0.02	43	P2-6-LE2	2.39 ± 0.4
9	P1N-6-LE2	1.37 ± 0.2	44	P2-5-LE4	2.01 ± 0.3
10	P1N-5-LE1	4.18 ± 0.9	45	P2-8-PE4	4.35 ± 0.8
11	P1N-5-LE2	0.25 ± 0.03	46	P2-8-PE3	1.73 ± 0.2
12	P1-13-PE4	0.37 ± 0.03	47	P1-5-LE1	1.08 ± 0.2
13	P1-4-LE3	5.57 ± 0.9	48	P2-8-PE1	1.41 ± 0.3
14	P1-13-PE3	0.20 ± 0.02	49	P1N-1-LE1	0.16 ± 0.02
15	P2-2-LE1	2.79 ± 0.3	50	P1N-1-LE2	0.08 ± 0.01
16	P1-1-LE1	8.99 ± 1.2	51	P1N-1-LE3	0.20 ± 0.04
17	P1-2-LE1	10.89 ± 1.5	52	P1N-1-LE4	0.09 ± 0.02
18	P1-13-PE2	0.44 ± 0.04	53	P1N-1-LE6	1.16 ± 0.2
19	P2-12-BE1	3.88 ± 0.4	54	P1N-10-LE2	0.42 ± 0.1
20	P1N-7-LE1	4.51 ± 0.4	55	P2-6-LE2	0.18 ± 0.03
21	P1N-3-LE1	1.91 ± 0.1	56	P1-13-PE1	19.98 ± 1.7
22	P1N-4-LE2	2.75 ± 0.3	57	P2-12-BE4	3.35 ± 0.8
23	P1-2-LE2	2.12 ± 0.2	58	P2-2-LE4	0.13 ± 0.03
24	P1N-4-LE1	1.14 ± 0.1	59	P1-3-E4	0.13 ± 0.02
25	P1N-6-LE1	7.47 ± 0.8	60	P2-15-SE3	0.45 ± 0.1
26	PIN-7-LE4	1.67 ± 0.1	61	P2-15-SE4	0.23 ± 0.04
27	P1-3-LE1	2.46 ± 0.4	62	P2-15-SE2	5.41 ± 0.9
28	P1N-12-LE1	3.62 ± 0.3	63	P2-15-SE1	0.29 ± 0.03
29	P1-8-LE2	0.67 ± 0.1	64	P2-10-BE2	0.30 ± 0.04
30	P1N-8-LE3	1.56 ± 0.2	65	P2-2-LE2	0.57 ± 0.03
31	P1-7-LE3	4.40 ± 1.2	66	P1N-1-LE5	0.10 ± 0.03
32	P1-5-LE3	5.75 ± 0.9	67	P1N-1-LE7	0.51 ± 0.1
33	P2-9-PE1	4.57 ± 0.9	68	P1N-1-LE8	0.88 ± 0.2
34	P1N-9-LE2	5.10 ± 1.1	69	P5-2-LE1	2.28 ± 0.7
35	P2-9-PE2	3.91 ± 0.8	70	P5-2-LE2	0.72 ± 0.1

Supplementary Table S1 (continued).

S. No	Endophyte	Camptothecin yield (μg/g)
71	P6-3-LE1	3.21 ± 0.8
72	P5-5-LE3	0.96 ± 0.2
73	P4-2-LE2	0.20 ± 0.03
74	P4-2-LE3	6.45 ± 0.9
75	P5-4-LE3	1.62 ± 0.3
76	P6-5-BE1	0.14 ± 0.03
77	P6-5-BE2	0.56 ± 0.1
78	P6-5-BE3	0.83 ± 0.1
79	P4-4-LE1	52.31 ± 12.6
80	P6-2-SE2	9.40 ± 0.8
81	P6-2-SE3	0.81 ± 0.1
82	P4-4-LE2	405.50 ± 34.7
83	P4-4-LE3	1.67 ± 0.2
84	P5-1-LE1	1.50 ± 0.3
85	P5-4-LE1	73.73 ± 9.5
86	P4-2-LE1	7.45 ± 0.9
87	P4-1-LE3	0.75 ± 0.1
88	P6-4-BE2	0.67 ± 0.1
89	P4-1-LE1	253.67 ± 35.8
90	P4-3-LE3	1.11 ± 0.2
91	P4-6-PE2	445.05 ± 41.3
92	P5-3-LE2	0.83 ± 0.2
93	P6-1-SE2	40.95 ± 3.5
94	P4-5-LE2	1.90 ± 0.3

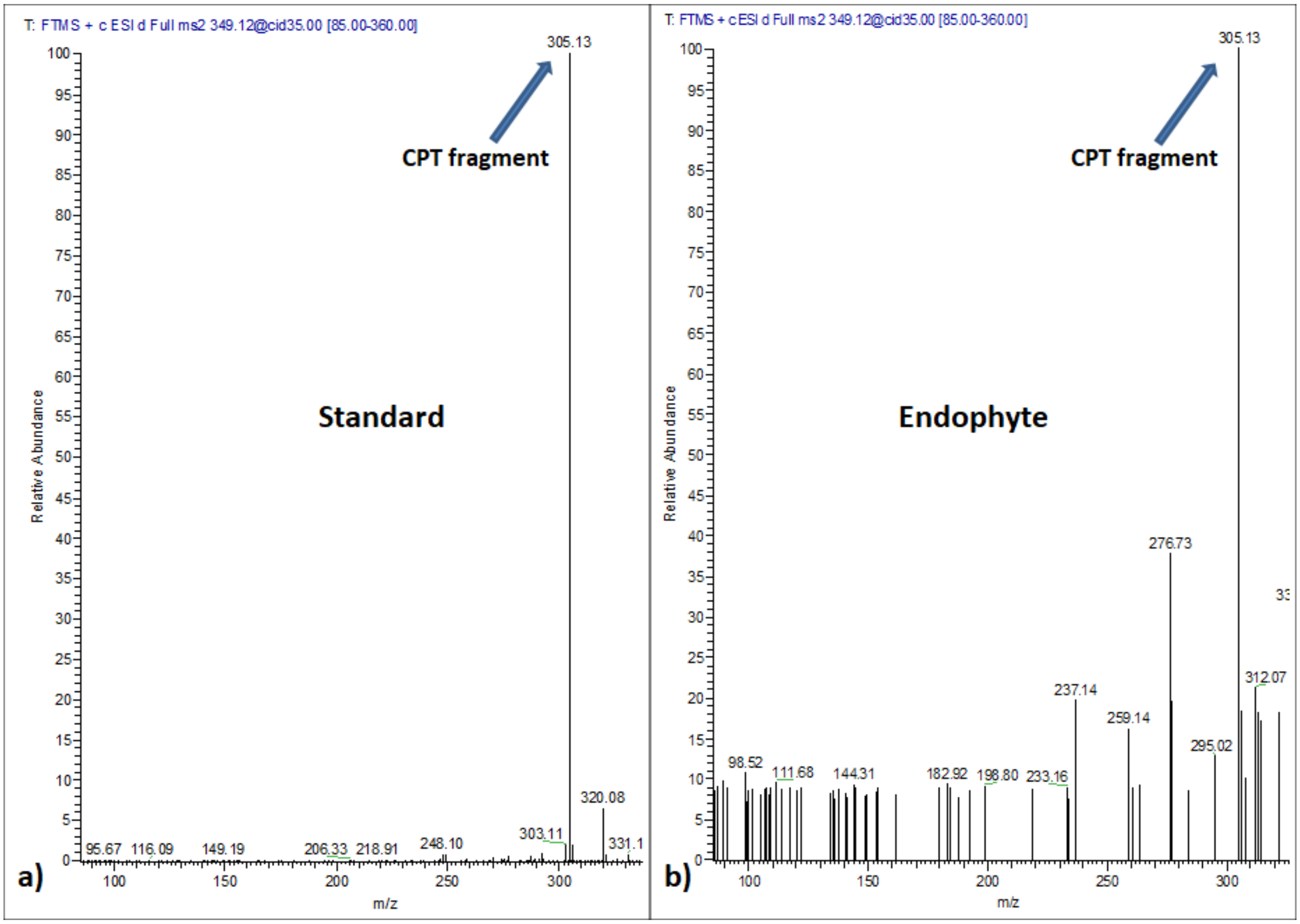

Supplementary Figure S1. Structural confirmation of camptothecin by collision induced dissociation in mass spectrometry. Fragment peak of m/z 305.13 witnessed in both the standard (a) and the isolated endophyte (b).

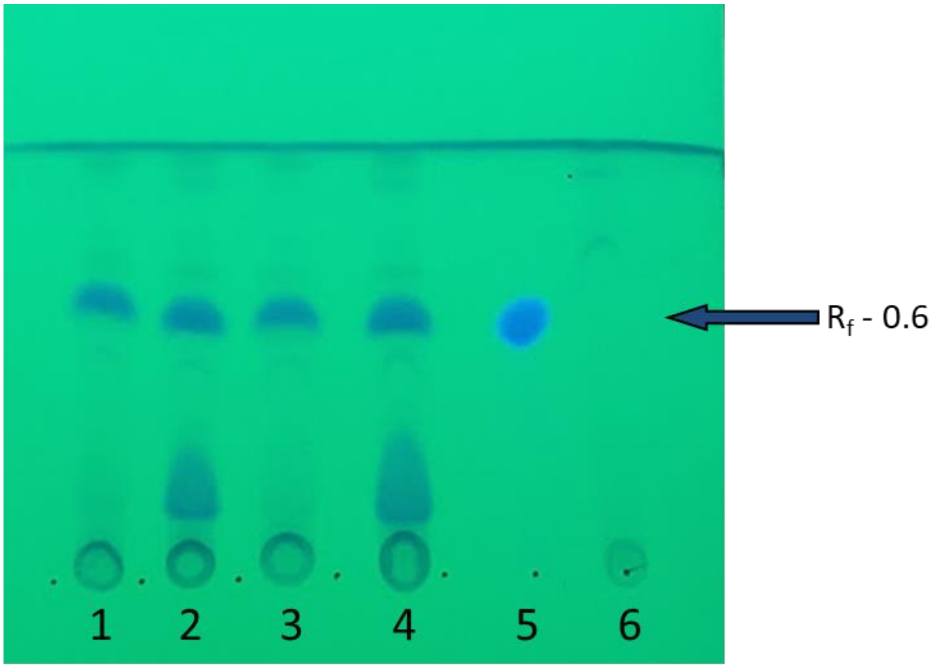

Supplementary Figure S2. Endophytes demonstrating camptothecin presence in TLC plates with an R_f value of 0.6 under 254 nm UV light. Camptothecin producing endophytic extract of P4-6-PE2 (Lane 1), P4-4-LE2 (Lane 2), P4-1-LE1 (Lane 3) and P5-4-LE1 (Lane 4); Standard camptothecin (Lane 5); Non camptothecin producing endophytic extract (Lane 6)

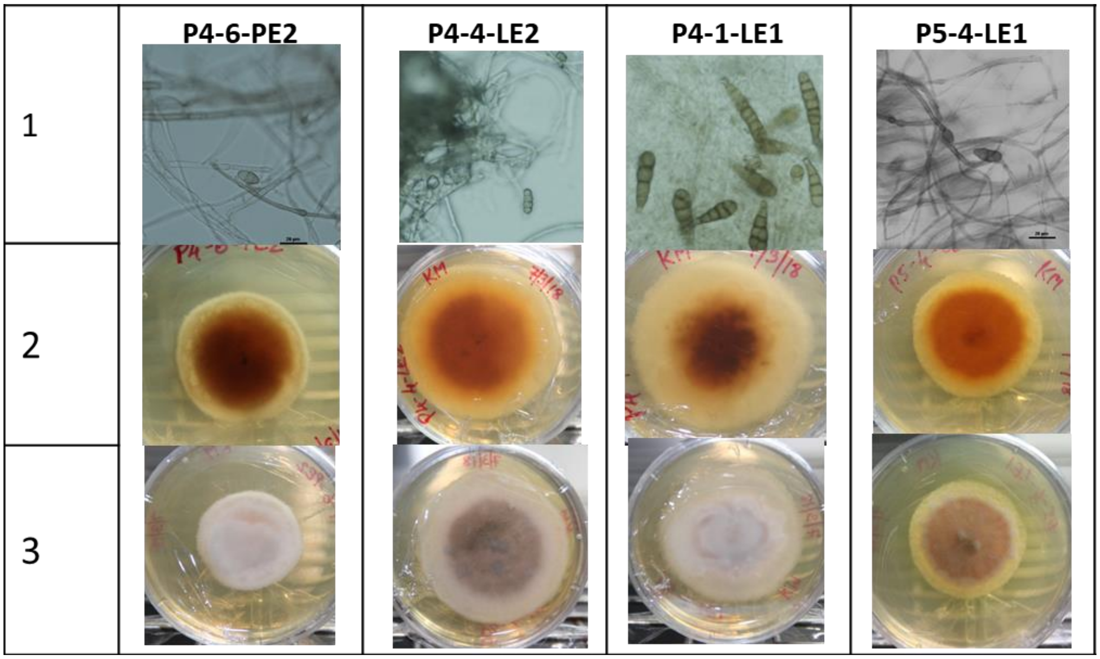

Supplementary Figure S3. Microscopic view of the top 4 camptothecin producing endophytes (Row 1) and morphological appearance of the isolated high camptothecin yielding endophytes from the bottom (Row 2) and top view (Row 3) on plates made with potato dextrose agar (PDA) medium.

Supplementary Table S2. Masses of ^{13}C labelled camptothecin molecules and their corresponding fragment ions after the removal of labelled and unlabeled CO_2, that could be visualized in the D-[U-^{13}C]-glucose fed culture extract.

**Possible Camptothecin with labelled carbons**	**Expected precursor m/z**	**Presence in spectra**	**Expected fragment m/z after removal of $^{13}CO_2$**	**Presence in spectra**
$C_{20}H_{16}N_2O_4$	349.12	ND	-	-
$^{13}C_1C_{19}H_{16}N_2O_4$	350.12	ND	305.13	ND
$^{13}C_2C_{18}H_{16}N_2O_4$	351.12	+	306.13	ND
$^{13}C_3C_{17}H_{16}N_2O_4$	352.13	+	307.13	ND
$^{13}C_4C_{16}H_{16}N_2O_4$	353.13	+	308.14	ND
$^{13}C_5C_{15}H_{16}N_2O_4$	354.13	+	309.14	ND
$^{13}C_6C_{14}H_{16}N_2O_4$	355.14	+	310.14	ND
$^{13}C_7C_{13}H_{16}N_2O_4$	356.14	+	311.15	ND
$^{13}C_8C_{12}H_{16}N_2O_4$	357.14	+	312.15	ND
$^{13}C_9C_{11}H_{16}N_2O_4$	358.15	+	313.15	+
$^{13}C_{10}C_{10}H_{16}N_2O_4$	359.15	+	314.16	+
$^{13}C_{11}C_9H_{16}N_2O_4$	360.15	+	315.16	+
$^{13}C_{12}C_8H_{16}N_2O_4$	361.15	ND	316.16	ND
$^{13}C_{13}C_7H_{16}N_2O_4$	362.16	+	317.16	+
$^{13}C_{14}C_6H_{16}N_2O_4$	363.16	+	318.17	+
$^{13}C_{15}C_5H_{16}N_2O_4$	364.16	+	319.17	+
$^{13}C_{16}C_4H_{16}N_2O_4$	365.17	+	320.17	+
$^{13}C_{17}C_3H_{16}N_2O_4$	366.17	+	321.18	+
$^{13}C_{18}C_2H_{16}N_2O_4$	367.17	+	322.18	+
$^{13}C_{19}C_1H_{16}N_2O_4$	368.18	+	323.18	+
$^{13}C_{20}H_{16}N_2O_4$	369.18	+	324.19	ND

[#]ND – Not detected

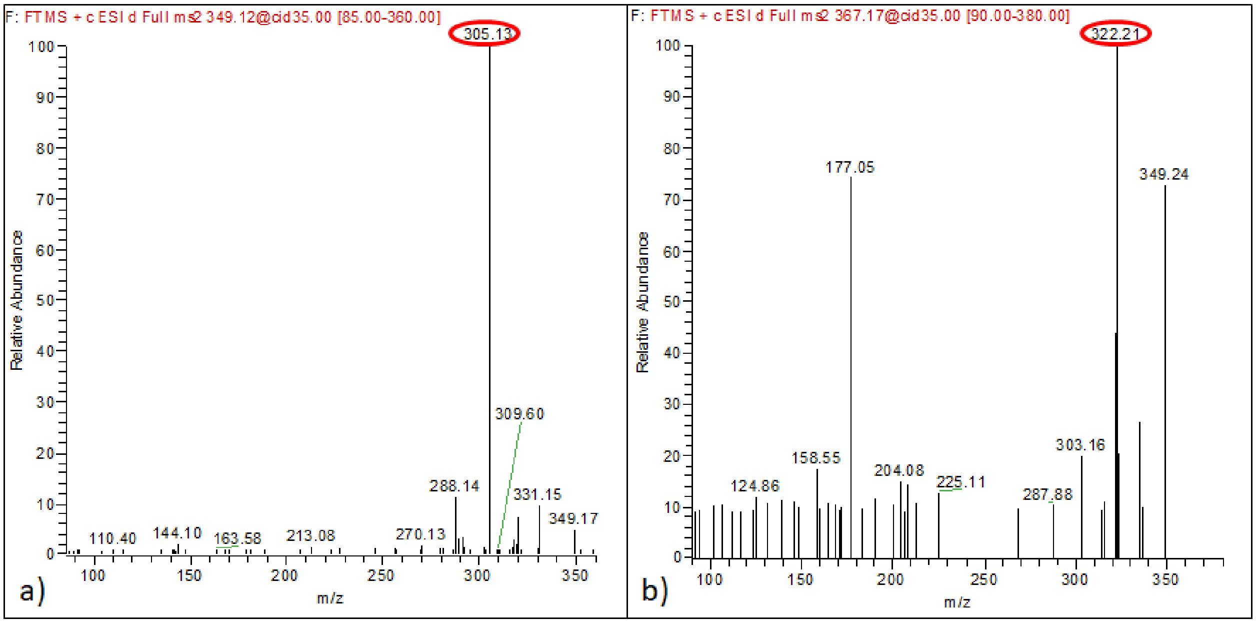

Supplementary Figure S4. Verification of camptothecin biosynthesis in *A. burnsii* at MS2 level in D-[U-^{13}C]-glucose study. Fragment peak after removal of CO_2 (44 Da) witnessed in the D-glucose fed camptothecin extract (a) and removal of $^{13}CO_2$ (45 Da) was witnessed in D-[U-^{13}C]-glucose fed camptothecin extract (b).

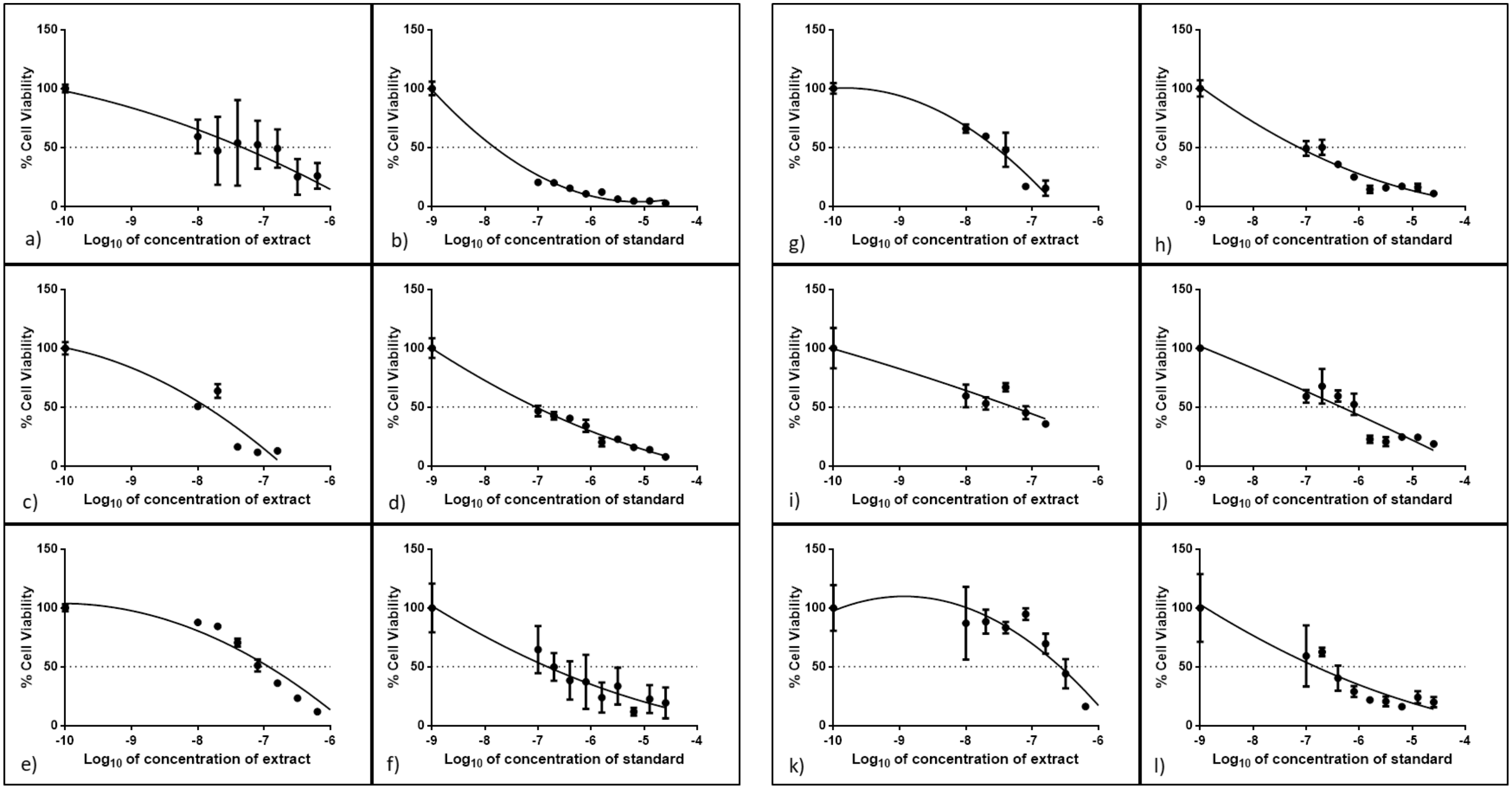

Supplementary Figure S5. Effect of camptothecin (in extract and standard) on cell viability. Cytotoxic effects of camptothecin extract and the camptothecin standard at varying concentrations on MCF7 (a, b), H1299 (c, d), SKOV3 (e, f), Caco-2 (g, h), HT29 (i, j), and HEK293T (k, l) cell lines cell line

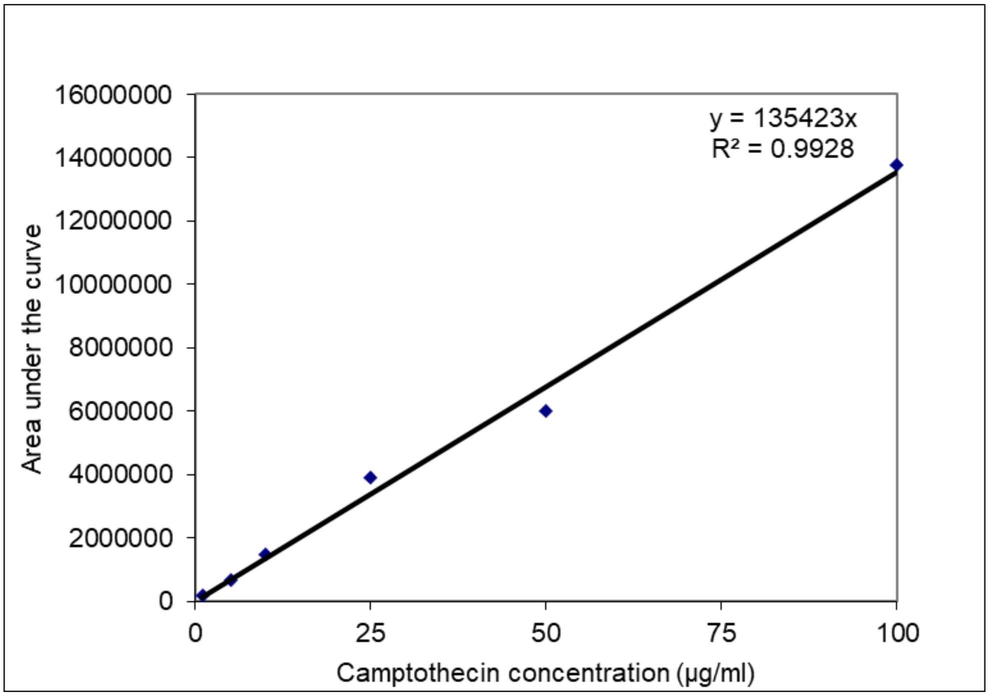

Supplementary Figure S6. The standard curve of camptothecin obtained by plotting various concentrations of camptothecin against their respective peak area from HPLC.

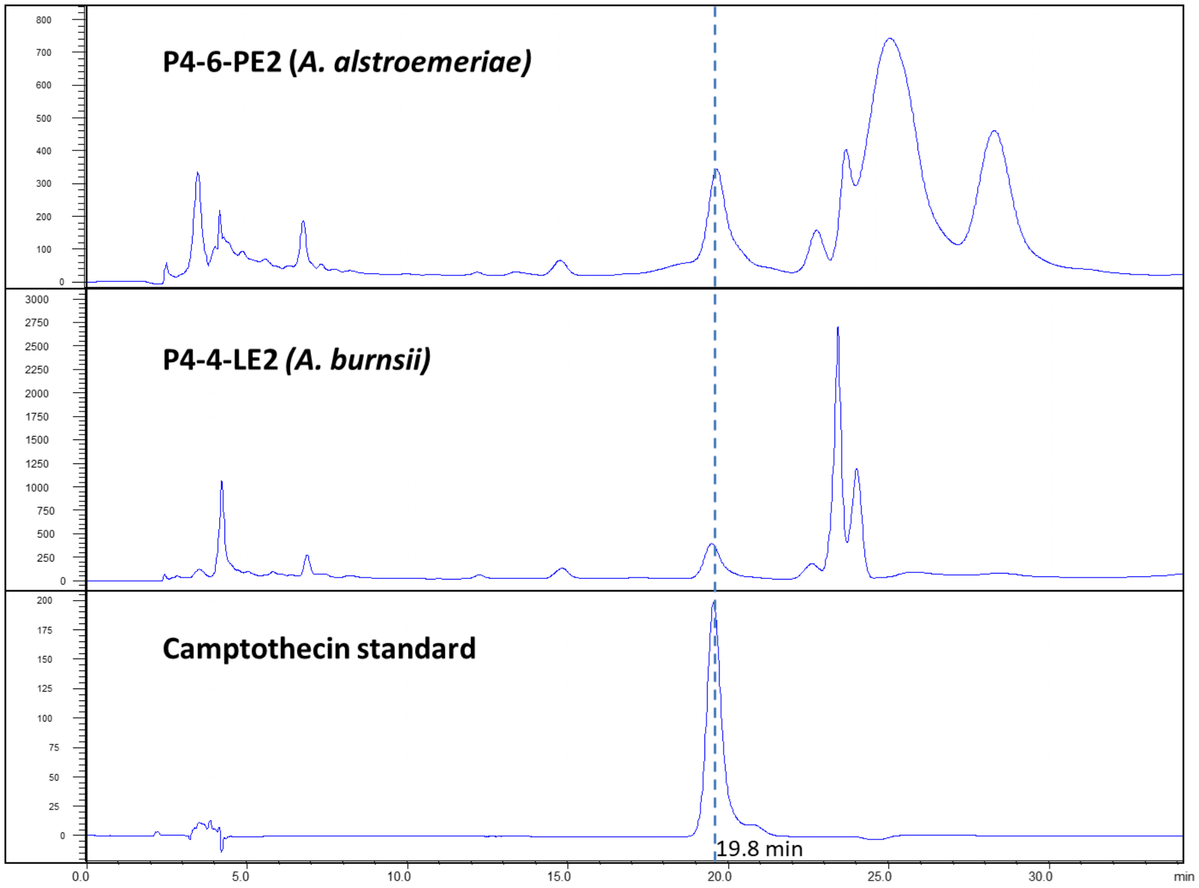

Supplementary Figure S7. The chromatograms obtained from P4-6-PE2 (*A. alstroemeriae*) (top) and P4-4-LE2 (*A. burnsii*) (middle) using HPLC, compared with that of standard camptothecin (bottom).

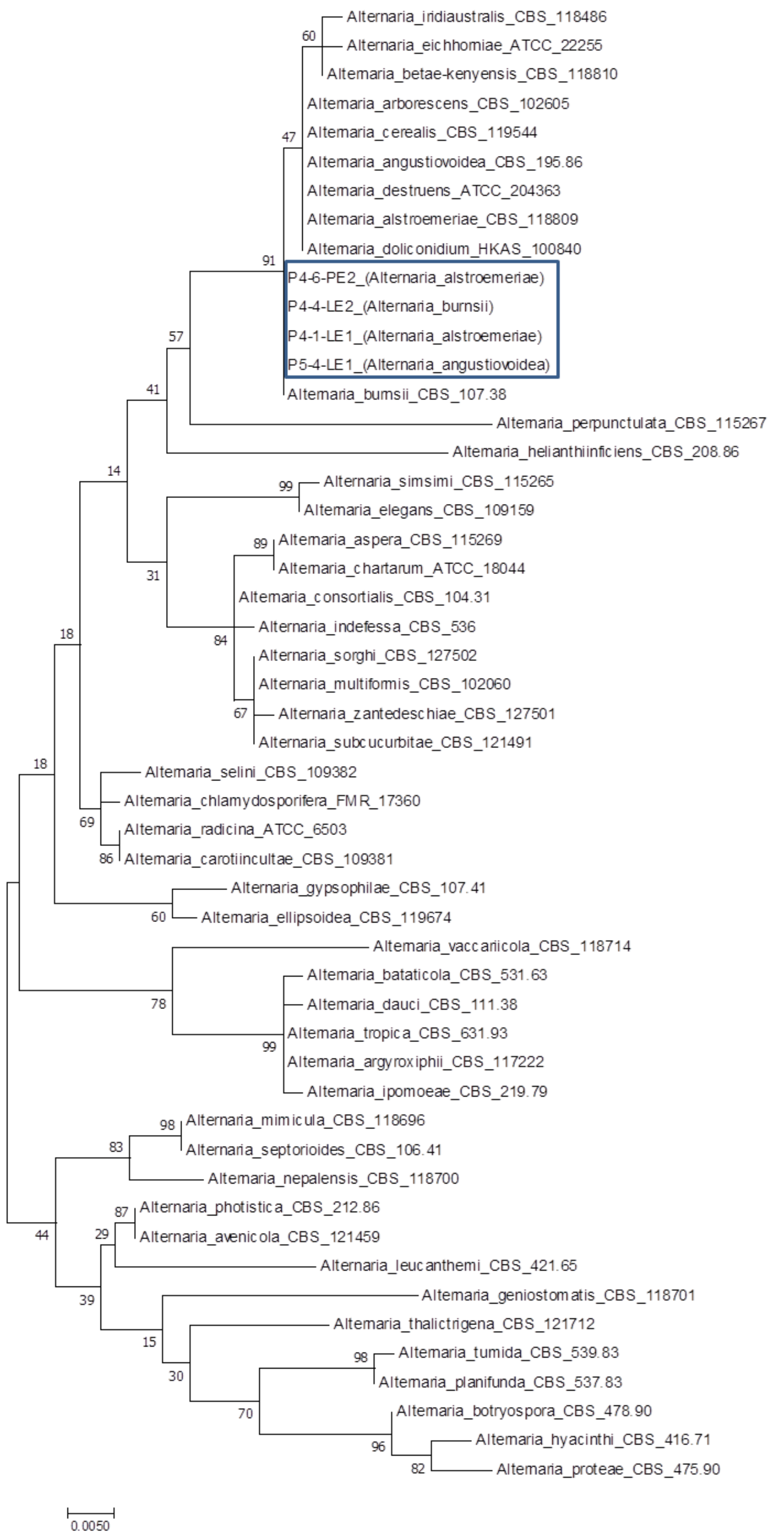

Supplementary Figure S8. Molecular Phylogenetic analysis by Maximum Likelihood method based on the Jukes-Cantor model [29]. The strains isolated in this study are shown

inside a blue rectangular box. The tree with the highest log likelihood (-1835.77) is shown. The percentage of trees in which the associated taxa clustered together is shown next to the branches. Initial tree(s) for the heuristic search were obtained automatically by applying Neighbor-Join and BioNJ algorithms to a matrix of pairwise distances estimated using the Maximum Composite Likelihood (MCL) approach, and then selecting the topology with superior log likelihood value. The tree is drawn to scale, with branch lengths measured in the number of substitutions per site. The analysis involved 51 nucleotide sequences. Codon positions included were 1st+2nd+3rd+Noncoding. All positions containing gaps and missing data were eliminated. There were a total of 471 positions in the final dataset. Evolutionary analyses were conducted in MEGA7 [30].
